# Association between birth by caesarian section and anxiety, self-harm: a gene-environment interaction study using UK Biobank data

**DOI:** 10.1186/s12888-023-04720-0

**Published:** 2023-04-07

**Authors:** Yumeng Jia, Shiqiang Cheng, Li Liu, Bolun Cheng, Chujun Liang, Jing Ye, Xiaomeng Chu, Yao Yao, Yan Wen, Om Prakash Kafle, Feng Zhang

**Affiliations:** Key Laboratory of Trace Elements and Endemic Diseases of National Health and Family Planning Commission, School of Public Health, Health Science Center, Xi’an Jiaotong University, Xi’an, 710061 China

**Keywords:** Anxiety, Self-harm, Birth by caesarian section, Genome-wide by environment interaction study

## Abstract

**Background:**

Limited efforts have been paid to explore the underlying genetic mechanisms of birth by caesarian section (CS) affecting the risks of adult anxiety and self-harm.

**Methods:**

Using UK Biobank cohort, the logistic regression model was first applied to evaluate the associations of adult anxiety and self-harm with birth by CS. Using birth by CS as exposure variables, genome-wide by environment interaction study (GWEIS) was then applied by PLINK2.0 to identify associated genes interacting with birth by CS for anxiety and self-harm.

**Results:**

In the observational study, significant associations were observed between birth by CS and anxiety (odds ratio (OR) = 1.24; 95% confidence interval (CI), 1.12–1.38; *P* = 4.86 × 10^− 5^), and self-harm (OR = 1.12; 95% CI, 1.01–1.24; *P* = 2.90 × 10^− 2^). GWEIS revealed multiple suggestive genes interacted with birth by CS for anxiety, such as *DKK2* (rs13137764, *P* = 1.24 × 10^− 9^, adjusted *P* = 2.68 × 10^− 7^) and *ATXN1* (rs62389045, *P* = 4.38 × 10^− 8^, adjusted *P* = 3.55 × 10^− 6^). For self-harm, significant gene-environment interactions of birth by CS on self-harm were detected, such as *ALDH1A2* (rs77828167, *P* = 1.62 × 10^− 8^; rs116899929, *P* = 1.92 × 10^− 8^) and *DAB1* (rs116124269, *P* = 3.20 × 10^− 8^; rs191070006, *P* = 3.63 × 10^− 8^).

**Conclusions:**

Our results suggested that birth by CS was associated with the risk of adult anxiety and self-harm. We also discovered some genes interacted with birth by CS might influence the risk of anxiety and self-harm, which may provide novel clues for the pathogenesis of those mental disorders.

**Supplementary Information:**

The online version contains supplementary material available at 10.1186/s12888-023-04720-0.

## Background

Mental disorder is a behavioral or mental pattern that causes significant distress or impairment of personal functioning [1]. According to a recent study, the estimated global burden of mental illness accounts for 32.4% of years lived with disability (YLDs) and 13.0% of disability-adjusted life-years (DALYs), placing a heavy burden on global public health [2]. Anxiety is one of the common but serious mental disorders, with an estimated global prevalence of 7.3% [3]. Besides, it is the sixth leading cause of disability in high-income and low-income countries [4]. Self-harm is one of the main public health issues with a symptom of mental and personality disorders, characterized by cutting, burning, or scratching the skin, or by drug overdose without the intention of committing suicide [5]. The World Health Organization estimates that, as of 2010, 880,000 deaths occur as a result of self-harm [6]. Therefore, it is essential to identify and prevent mental illness in high-risk populations for reducing social burden and improving mental health in adults.

Genetic factors are demonstrated to play an important role in the development of common mental disorders. In a large population-based cohort (N = 122 k individuals) genome-wide association study, three independent loci (on chromosomes 9, 11 and 13) were identified to significantly associated with suicidality [7]. In addition, non-suicidal self-harm was moderately heritable and identified shared genetic factors between self-harm and suicide [8, 9]. Twin studies suggested 30–40% genetic influence on individual differences in anxiety [10]. However, the genetic mechanism of mental illness remains elusive up to now. The predominant view until now is that biological, psychological, and environmental factors and their interactions all contribute to the development or progression of mental disorders [11]. Compared with previous approaches, by taking environmental factors into account, genome-wide by environment interaction study (GWEIS) is now becoming a popular method to explore disease-associated genetic variations that interact with environmental risk factors. By conducting a GWEIS, recent studies have identified several SNPs with genome-wide significant GxE effects that are associated with depression, stress, or other psychiatric disorders [12, 13].

Caesarean section (CS), a surgery to deliver babies, can reduce the mortality of mothers, fetuses, and newborns to a certain extent. However, excessive selection of CS poses a threat to both short-term and long-term maternal and child health [14]. Offspring birth by CS are prone to nervous system diseases [14], including abnormal neuro-psychological development of offspring [15, 16]. A recent animal study observed that offspring birth by CS had anxiety-like and depression-like behaviors in adolescence and adulthood accompanied by lower 5-HT and 5-HIAA levels [17]. In addition, young adults birth by CS exhibit increased psychological distress and anxiety compared to age-matched young adults born vaginally [18]. However, limited efforts have been paid to explore the underlying genetic mechanisms of birth by CS affecting the risks of adult anxiety and self-harm. Besides, the World Health Organization (WHO) in 2004 recommended that the CS use should be less than 15% [19], whereas the number of CS is increasing globally since 1990 [20]. Clarifying the effects and identified the potential genetic variants of birth by CS on adult anxiety and self-harm may provide basis for policies and programs to prevent unnecessary CS, which will benefit the mental health in adults.

In this study, we aimed to explore the effects of birth by CS on adult anxiety and self-harm in UK Biobank cohort and to further investigate its underlying genetic mechanisms. First, we estimated the association of birth by CS with the risk of anxiety and self-harm through a logistic regression model. GWEIS was then applied to explore the genetic variation interaction between birth by CS and the risk of anxiety and self-harm. Our study holds the potential for clarifying the functional relevance of birth by CS with adult anxiety and self-harm.

## Methods

### Ethical approval

UK Biobank has electronic signed consent from the study participants and ethical approval was obtained from Northwest Multi-Center Research Ethics Committee (reference 11/NW/0382) and has been conducted in accordance with the ethical standards, according to the Declaration of Helsinki, and according to national and international guidelines. All patients gave written informed consent to participate after verbal and written information.

### UK Biobank dataset

The UK Biobank study is a large prospective cohort study that included health, hospital records and genetic data from 502,656 participants aged 40–69 in 2006 and 2010 [21]. We used the imputed genotype dataset made available by UK Biobank in its July 2017 release. We restricted participants to “white British” individuals based on self-reported ethnicity. Subjects who had a self-reported gender inconsistent with the genetic gender, who were genotyped but not imputed or who withdraw their consents were removed. Participants who are identified with outliers in heterozygosity and missing rate were excluded (data field 20,027). All participants agreed to use their anonymous data to conduct any health-related studies and to reconnect for further sub-studies.

Genotyping, quality control and imputation were performed by the UK Biobank. DNA samples of all participants in the UK Biobank were genotyped using either the Affymetrix UK BiLEVE (807,411 markers) or Affymetrix UK Biobank Axiom (825,927 markers) array [22]. SNPs were imputed by IMPUTE2 against the reference panel of the Haplotype Reference Consortium, 1000 Genomes and UK10K projects. Full details regarding these data are available elsewhere [23]. This research has been conducted using the UK Biobank Resource under Application Number 46,478. The authors thank all UK Biobank participants and researchers who contributed or collected data.

### Phenotypes definition

Birth by CS was collected from the response to the UK Biobank online “Thoughts and Feelings” digestive health questionnaire: “Were you birth by caesarian section?” by choosing “Do not know (-121)”, “No (0)”, “Yes (1)” and “Prefer not to answer (-818)”. The subjects whose answers are “Do not know (-121)” and “Prefer not to answer (-818)” were excluded from this study.

The case group criteria of anxiety were defined self-reported according to two UK Biobank fields: 20,002 and 20,544. Anxiety was selected based on the code 1287 from ID 20,002 and code 15 from ID 20,544 as cases. In order to obtain a comprehensive and accurate control group, we strictly set the control group threshold by Davis et al. research [24], which is based on Patient Health Questionnaire (PHQ-9), general anxiety disorder (GAD-7) [25] and another strict criterion based on composite international diagnostic interview short-form (CIDI-SF) [25, 26]. For the control group of anxiety, after excluding the anxiety defined in our study and generalized anxiety disorder (GAD) ever defined in Davis et al. research, we chose the participants who did not endorse anxiety or screen positive on GAD-7. More precisely, participants whose GAD score < 5. GAD-7 is a classification algorithm with a total score (0–21) used to screen for and measure anxiety severity, focusing on seven anxious symptoms and signs (as detailed below: Feeling nervous, anxious or on edge 20,506, Not being able to stop or control worrying 20,509, Worrying too much about different things 20,520, Trouble relaxing 20,515, Being so restless that it is hard to sit still 20,516, Becoming easily annoyed or irritable 20,505, Feeling afraid as if something awful might happen 20,512. In order to meet the 0–3 score for each item of GAD, the 7 symptom scores (1–4) of our team UK were all reduced by 1 point, which was then added up and participants with GAD score < 5 were selected.

According to the previous study [7], self-harm phenotype was also defined using a touch screen questionnaire according to two UK Biobank fields: 20,480 and 20,485. Participants were asked, “Have you deliberately harmed yourself, whether or not you meant to end your life?” and “Have you contemplated harming yourself (for example by cutting, biting, hitting yourself or taking an overdose)?“. “Prefer not to answer” was set to “missing” in our analyses. Participants who answered both questions “NO” would be classified as the control group, and one or two “YES” would be classified as the case group. Participants who have attempted or completed suicide were excluded from the control group of self-harm analyses.

### Observational analyses

The associations between birth by CS and anxiety, self-harm behavior were estimated using a logistic regression model. The exposures variable was birth by CS and the outcome variables were anxiety and self-harm. Sex, age, cigarette smoking, alcohol drinking and the first 10 principle components of population structure were adjusted as covariates. Beta coefficient or odd ration (OR) with 95% confidence intervals (CI) and p-values were calculated by the logistic regression model. All statistical analyses were conducted by R 3.5.3 (https://www.r-project.org/).

### Genome-wide by environmental interaction analysis

GWEIS was conducted to explore the interaction between SNP and birth by CS in mental disorders in UK Biobank cohort by using the PLINK 2.0 function “glm”, with the following formula:

$$logit\left[ {P\left( {D = {\rm{ }}1|G,{\rm{ }}E} \right)} \right]{\rm{ }} = {\beta _0} + {\beta _g}G + {\beta _e}E + {\beta _{ge}}GE$$

Let *D* denotes the disease outcome, where *G* denotes genetic factors and *E* denotes environmental factors [27]. Specific in this study, the function used a logistic model to evaluate the association because the outcome variables are binary variable. The outcome variables, including anxiety and self-harm behavior, were adjusted by sex, age, cigarette smoking, alcohol drinking and the first 10 principle components of population structure. According to the previous study [28, 29], additional quality control was also performed to select high-quality SNPs as follows: SNPs with low call rate (< 0.95), low Hardy-Weinberg equilibrium exact test P values (< 0.001) and low minor allele frequencies (MAFs; < 0.01) were excluded. To investigate the role of birth by CS, we evaluated the interaction effects of additive (ADD)×birth by CS model for each SNP on anxiety and self-harm, respectively. SNPnexus (https://www.snp-nexus.org/v4/) was used to investigate the location of the SNPs to identify the associated genes and for the functional annotation [30]. Systematic inflation (or deflation) of test statistics was quantified by the overall behavior of genome-wide test statistics [31]. The deviation of the estimated λ from one indicates that test statistics are problematic, either due to population stratification or cryptic relatedness [31]. A significant threshold was set at *P* = 5.0 × 10^− 8^ for genome-wide by environment interaction effects. The Manhattan plots were generated using FUMA [32] (found at https://fuma.ctglab.nl/).

### LocusZoom plots

LocusZoom plots (Additional file 2 and file 3) were created using the LocusZoom tool [33] (found at http://locuszoom.sph.umich.edu/locuszoom/) by uploading summary statistics from the birth by caesarean section and anxiety and self-harm GWEIS, respectively.

## Results

### Basic characteristics of study samples

A total of 83,615 participants completed the anxiety-related questions including 47,554 were female and the mean (SD) age was 56.42 (7.48) years old. 14,556 participants were classified into case group, among which 472 were birth by CS and 14,094 were not birth by CS. A total of 96,492 participants answered the self-harm-related questions including 56,081 were female and the mean (SD) age was 56.21 (7.54) years old. 14,445 participants were classified into case group, among which 471 were birth by CS and 13,947 were not birth by CS (Table 1).

**Table 1:** Basic characteristics of study samples from UK Biobank

		Birth by CS (yes/no)
**Anxiety**	Case	14,566 (472/14,094)
	Control	69,049 (1,788/67,261)
	Sex (Female)	47,554
	Age ± SD	56.42 ± 7.48
**Self-harm**	Case	14,445 (471/13,974)
	Control	82,017 (2,167/79,850)
	Sex (Female)	56,081
	Age ± SD	56.21 ± 7.54

Note: CS, caesarean section; SD, standard deviation

### Association between birth by CS and mental disorders

Due to the imbalances in age and sex for both anxiety and self-harm between cases and controls, sex and age were adjusted as covariates (Additional file 1). In UK Biobank cohort, significant associations were observed between birth by CS and anxiety (odds ratio (OR) = 1.24; 95% confidence interval (CI), 1.12–1.38; *P* = 4.86 × 10^− 5^), and self-harm (OR = 1.12; 95% CI, 1.01–1.24; *P* = 0.029) (Table 2).

**Table 2:** Association between anxiety, self-harm behavior and birth by CS

Instrument	Outcome	Statistics	SE	OR (95% CI)	*P*
**Birth by CS**	Anxiety	4.06	0.05	1.24 (1.12–1.38)	4.86 × 10^− 5^
	Self-harm	2.18	0.05	1.12 (1.01–1.24)	2.90 × 10^− 2^

Note: CS, caesarean section; SE, standard error; CI, confidence interval; OR, odd ratios

### GWEIS results

**Fig. 1:**
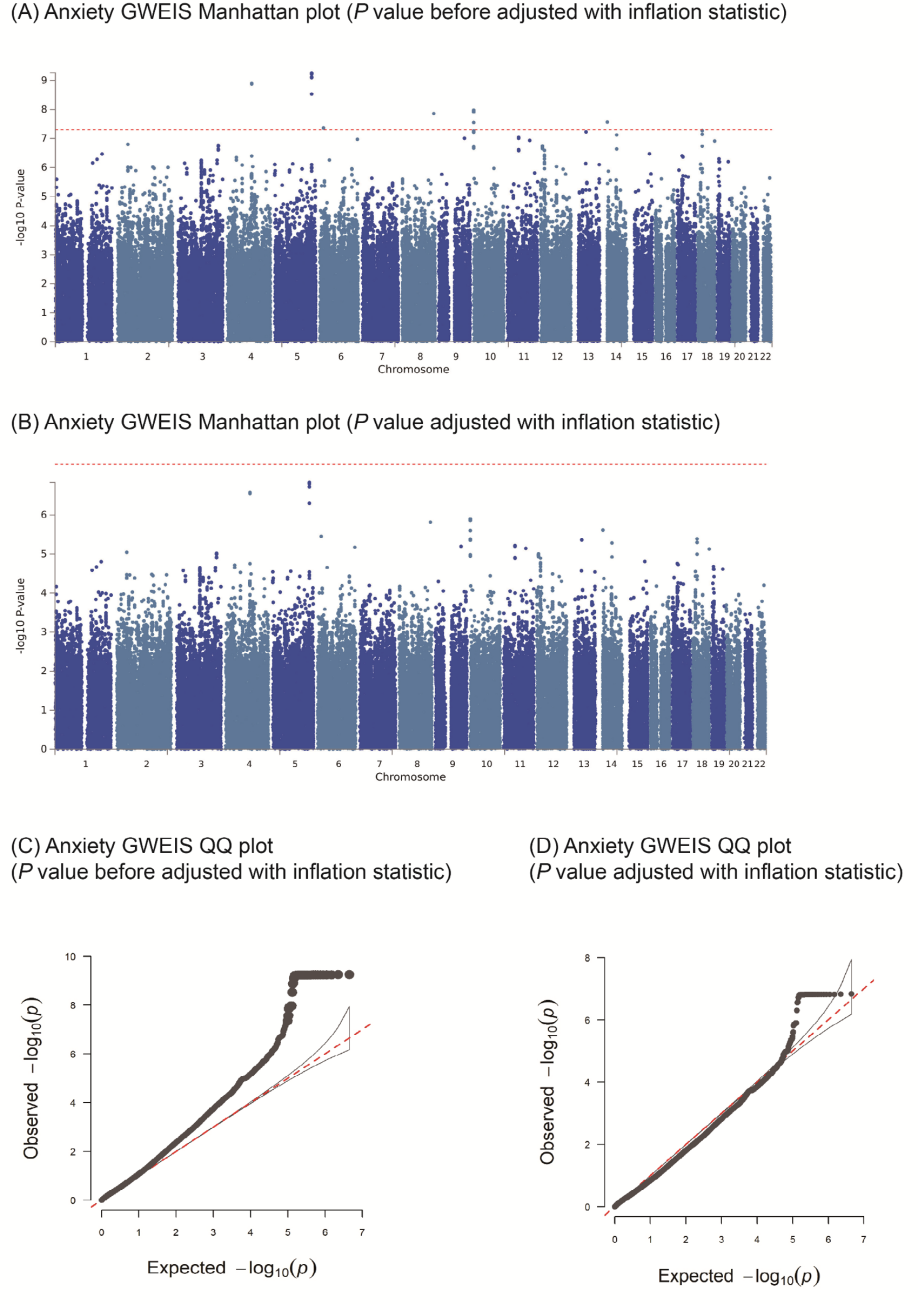
Manhattan and quantile-quantile (QQ) plots of GWEIS between birth by caesarean section and anxiety. **(A)** The Manhattan plot of GWEIS for anxiety (*P* value before adjusted with inflation statistic) and **(B)** The Manhattan plot (*P* value adjusted with inflation statistic) of GWEIS for anxiety show association test (-log_10_*P* value on the y-axis against physical autosomal location on the x-axis). The standard genome-wide significance cut-off of *P* = 5 × 10^− 8^ is shown by the horizontal red line. **(C)** The QQ (quantile-quantile) plot of GWEIS for anxiety (*P* value before adjusted with inflation statistic). **(D)** The QQ (quantile-quantile) plot of GWEIS for anxiety (*P* value adjusted with inflation statistic)

The degree of inflation for anxiety was λ_GC anxiety_ = 1.18, showing some bias, so we performed genomic control to adjust *P* value. We identified a total of 45 suggestive SNPs interacting with birth by CS, such as *DKK2* (rs13137764, *P* = 1.24 × 10^− 9^, adjusted *P* = 2.68 × 10^− 7^; rs13148189, *P* = 1.34 × 10^− 9^, adjusted *P* = 2.83 × 10^− 7^), rs62389045 located in *ATXN1* (*P* = 4.38 × 10^− 8^, adjusted *P* = 3.55 × 10^− 6^), and rs62522074 located in *COL22A1* (*P* = 1.39 × 10^− 8^, adjusted *P* = 1.54 × 10^− 6^). In addition, seven SNPs occur near in *DIP2C*, including rs61831032 (*P* = 1.06 × 10^− 8^, adjusted *P* = 1.27 × 10^− 6^) were identified as suggestive SNPs. (Fig. 1; Table 3 and Additional file 2).

**Table 3:** Interactions between suggestive individual SNPs and birth by caesarian section in anxiety

CHR	Position	Most significant SNP	Risk allele	Gene	SE	OR	*P*	Adjusted *P*
5	163,079,410 ~ 163,086,731	rs34416550	G		0.050	1.36	5.50 × 10^− 10^	1.49 × 10^− 7^
4	107,872,868 ~ 107,883,586	rs13137764	G	*DKK2*	0.064	1.47	1.24 × 10^− 9^	2.68 × 10^− 7^
10	575,042 ~ 601,778	rs11814503	T	*DIP2C*	0.063	1.43	1.06 × 10^− 8^	1.27 × 10^− 6^
8	139,918,166	rs62522074	C	*COL22A1*	0.089	1.66	1.39 × 10^− 8^	1.54 × 10^− 6^
14	20,549,093	rs1959650	C		0.045	1.29	2.68 × 10^− 8^	2.49 × 10^− 6^
10	565,830	10:565830_ GCACACCCACA_G	G		0.066	1.44	2.79 × 10^− 8^	2.56 × 10^− 6^
6	16,617,181	rs62389045	C	*ATXN1*	0.142	2.17	4.38 × 10^− 8^	3.55 × 10^− 6^

Note: CHR, chromosome; SNP, single nucleotide polymorphism; SE, standard error; OR, odd ratios

**Fig. 2:**
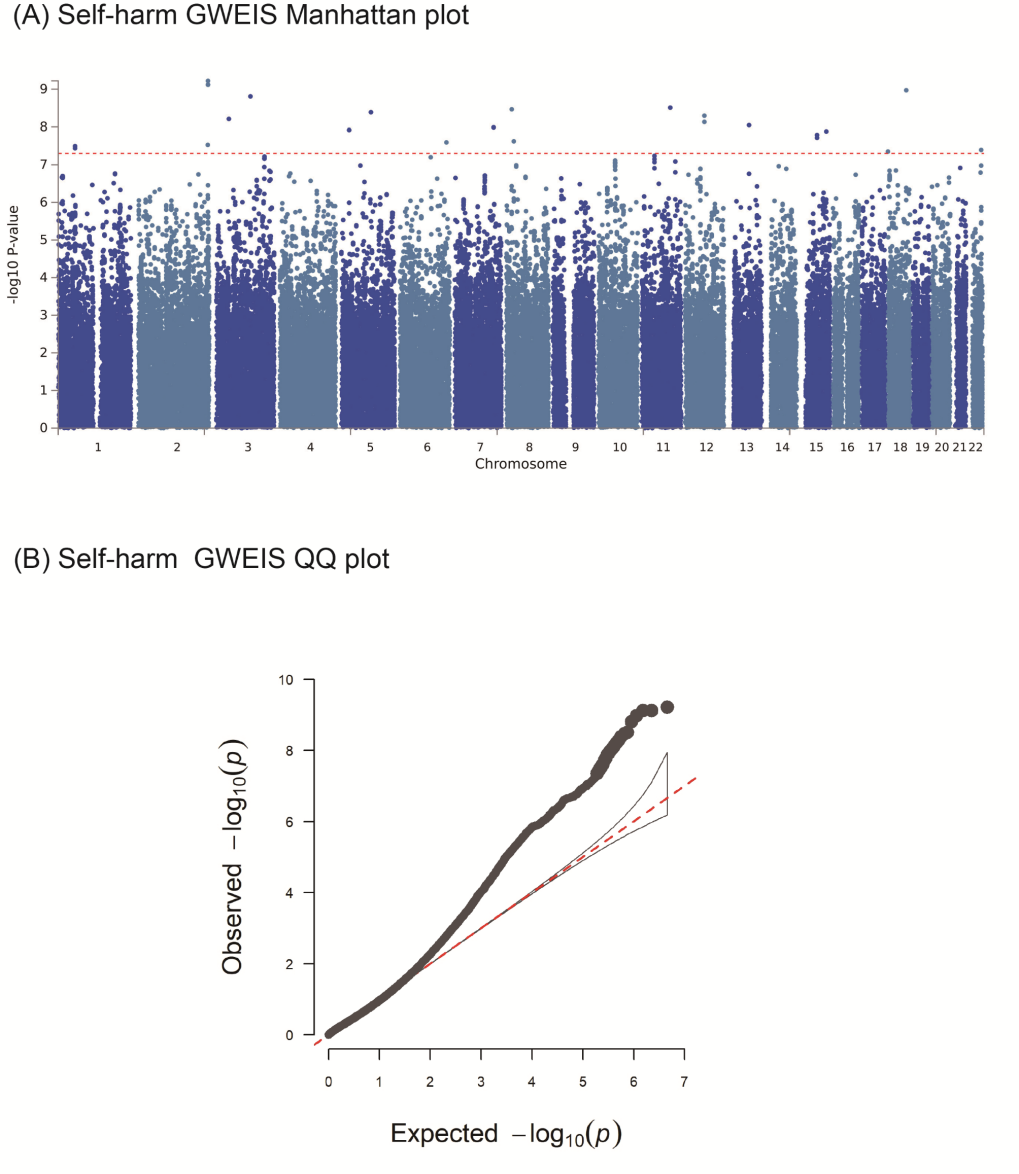
Manhattan and quantile-quantile (QQ) plots of GWEIS between birth by caesarean section and self-harm. **(A)** The Manhattan plot shows association test (-log_10_*P* value on the y-axis against physical autosomal location on the x-axis). The standard genome-wide significance cut-off of P = 5 × 10^− 8^ is shown by the horizontal red line. **(B)** The QQ (quantile-quantile) plot of GWEIS for self-harm

The degree of inflation for self-harm was λ_GC self−harm_ = 0.97, and we identified a total of 24 significant SNPs interacting with birth by CS at *P* < 5.0 × 10^–8^, such as *ALDH1A2* (rs77828167, *P* = 1.62 × 10^− 8^; rs116899929, *P* = 1.92 × 10^− 8^), *DAB1* (rs116124269, *P* = 3.20 × 10^− 8^; rs191070006, *P* = 3.63 × 10^− 8^), and *LRRFIP1* (rs62194228, *P* = 6.02 × 10^− 10^; rs3806505, *P* = 7.57 × 10^− 10^; rs55874185, *P* = 7.62 × 10^− 10^). rs140171389 (*P* = 4.03 × 10^− 8^) occurs near in *CELSR1* and rs75563143(*P* = 4.45 × 10^− 8^) occurs near in *COLEC12* were also identified as significant SNPs. (Fig. 2; Table 4 and Additional file 3).

**Table 4:** Interactions between individual SNPs and birth by caesarian section in self-harm with P < 5 × 10^–8^

CHR	Position	Most significant SNP	Risk allele	Gene	SE	OR	*P*
2	238,673,302 ~ 238,676,449	rs62194228	A	*LRRFIP1*	0.066	1.50	6.02 × 10^− 10^
18	62,904,454	rs72933283	A		0.152	2.53	1.07 × 10^− 9^
3	116,878,599	rs189957265	T		0.200	3.35	1.55 × 10^− 9^
11	97,704,650	rs117077436	C		0.410	11.34	3.09 × 10^− 9^
8	19,903,238	rs144469617	A		0.151	2.44	3.43 × 10^− 9^
5	101,312,655	rs80178526	T		0.090	1.70	4.09 × 10^− 9^
12	65,933,386 ~ 65,966,791	rs77050423	G	*AC025419.1*	0.174	2.76	5.06 × 10^− 9^
3	42,996,401	rs183426309	C	*AC092042.3*	0.209	3.36	6.17 × 10^− 9^
13	71,676,877	rs567777268	T	*LINC00348*	0.184	2.89	8.98 × 10^− 9^
7	132,916,768	rs148128132	C		0.138	2.21	1.02 × 10^− 8^
5	26,804,600	rs185846886	G		0.156	2.44	1.20 × 10^− 8^
15	90,347,914	rs41276918	G	*ANPEP*	0.194	3.00	1.31 × 10^− 8^
15	58,676,884 ~ 58,677,908	rs77828167	T	*ALDH1A2*	0.201	3.11	1.62 × 10^− 8^
8	27,020,339	rs75717648	A		0.117	1.92	2.38 × 10^− 8^
6	159,442,296	rs115127002	G	*AL035530.2*	0.168	2.56	2.55 × 10^− 8^
2	237,951,305	rs72973905	A		0.175	2.65	2.96 × 10^− 8^
1	57,752,892 ~ 57,771,618	rs116124269	T	*DAB1*	0.125	2.00	3.20 × 10^− 8^
22	46,828,697	rs140171389	AT	*CELSR1*	0.295	5.05	4.03 × 10^− 8^
18	412,863	rs75563143	C	*COLEC12*	0.149	2.25	4.45 × 10^− 8^

Note: CHR, chromosome; SNP, single nucleotide polymorphism; SE, standard error; OR, odd ratios

## Discussion

In this study, we conducted an observational and GWEIS analysis to explore the relationship between birth by CS and adult anxiety and self-harm. We found significant associations between birth by CS and the risk of anxiety and self-harm respectively. In addition, GWEIS identified multiple genes that interacted with birth by CS for anxiety and self-harm.

It has been suggested by a previous systematic review and meta-analysis that birth by CS is associated with certain neurodevelopmental and psychiatric disorders [15, 34, 35], such as autism spectrum disorders and attention-deficit/hyperactivity disorders [15], and poor cognitive performance [35]. Few efforts have been paid to explore the underlying genetic mechanisms of birth by CS affecting the risks of adult anxiety and self-harm [17, 18]. Our study identified significant associations between birth by CS and its risk on anxiety (OR = 1.24; 95% CI, 1.12–1.38; *P* = 4.86 × 10^− 5^) and self-harm (OR = 1.12; 95% CI, 1.01–1.24; *P* = 2.90 × 10^− 2^) in UK Biobank cohort, suggesting that birth by CS significantly increases the risks for both anxiety and self-harm in adults. These findings underline the need for a politics and program approach in responding to requests for a planned CS when there are no apparent increased risks from vaginal delivery.

Our GWEIS identified a total of 45 significant SNPs interacting with birth by CS for anxiety (Table 3). Among which, rs62389045 located in *ATXN1* (*P* = 4.38 × 10^− 8^, adjusted *P* = 3.55 × 10^− 6^) showed the highest OR value of 2.17, implying this genetic variant exhibits the highest risk interacting with birth by CS for anxiety. *DKK2* rs13137764, *P* = 1.24 × 10^− 9^, adjusted *P* = 2.68 × 10^− 7^; rs13148189, *P* = 1.34 × 10^− 9^, adjusted *P* = 2.83 × 10^− 7^) and *ANTX1* were observed as significant genes for anxiety. *DKK2* is an important member of the *DKK* gene family. The *DKK* gene family is an ancient and evolutionarily conserved gene family [36]. In recent years, a large number of studies showed that *DKK* gene family plays an important role in embryonic development, neural regeneration, synaptogenesis and so on [37]. Therefore, its role in neuropsychiatric disorders, such as cognitive impairment and emotional disorders, has attracted increasing attention [37]. According to a previous study, *DKK2* converges on β-catenin using distinct transduction pathways required to activate Wnt/β-catenin signaling and induce neural crest cells [38]. Zhao et al. have demonstrated an anxiety-specific response and contribution of activated neural stem cells to chronic pain through Wnt/β-catenin signaling, which may be targeted for treating chronic pain- or other diseases-associated anxiety [39]. However, there are few studies about the effect of *DKK2* on anxiety. We found *DKK2* interacting with birth by CS for anxiety. Further in vivo and in vitro functional studies are needed on this effect.

Ataxin-1 (*ATXN1*), the gene mutated in spinocerebellar ataxia type 1 (SCA1), is another significant candidate genetic variant interacting with birth by CS for anxiety. Lu et al. performed a series of behavioral tests on the *ATXN1*–with its paralog ataxin 1–like (*ATXN1L*) conditional knockout mouse lines to assess general activity, anxiety, learning and memory, and social behavior [40]. In the elevated plus-maze test, conditional knockout mice spent more time in the open arm and less time in the closed arm than control mice which was possible a result of reduced anxiety [40]. According to a previous study that modeled early-life unpredictable stress in developing rats found enhanced levels of anxiety when tested in adulthood compared to control, non-stressed adult rats [41]. The results showed that these behavioral changes were associated with upregulated *ANTX1* gene within the amygdala [41].

GWEIS also identified a total of 24 significant SNPs interacting with birth by CS for self-harm with *P* < 5 × 10^–8^ and OR>1 (Table 4), suggesting all of the significant SNPs are the risk factors interacting with birth by CS for self-harm. Among which, both the *ALDH1A2* (rs77828167, *P* = 1.62 × 10^− 8^; rs116899929, *P* = 1.92 × 10^− 8^) and *DAB1* (rs116124269, *P* = 3.20 × 10^− 8^; rs191070006, *P* = 3.63 × 10^− 8^) showed the OR value greater than 2.00, implying remarkable risk factors for self-harm. rs117077436 (*P* = 3.09 × 10^− 09^) has the highest OR value of 11.34, but there is limited research focused on its risk for self-harm. According to a previous study, after nonfatal self-harm, adolescents and young adults were at a markedly elevated risk of suicide [42]. It has been reported that self-harm and suicide are the predominant causes of decreased survival in patients suffering from schizophrenia [43]. *ALDH1A2*, encodes an enzyme for astrocyte-derived retinoic acid, is a key neuronal morphogen with relevance for schizophrenia. For example, Wan et al. observed a positive association between *ALDH1A2* and schizophrenics in the Chinese population [44]. In a methylome-wide association study of schizophrenia, *ALDH1A2* was identified to be the second-most significant site [45]. *DAB1*, a key component of the Reelin pathway [46], is sufficient to induce behavioral deficits related to psychiatric disorders. A recent study revealed that *DAB1* conditional knockout mice showed hyperactivity, decreased anxiety-like behavior, and a deficit in spatial reference and working memory [47]. The results indicated that the Reelin-DAB1 signaling in the cortex can be an important molecular basis for the regulation of behaviors [47]. Teixeira et al. observed a causal relation between the downregulation of DAB1 protein levels during development and the structural and behavioral deficits associated with psychiatric diseases in the adult [48]. Other significant genetic variants such as *COL22A1* (rs62522074, *P* = 1.39 × 10^− 8^, adjusted *P* = 1.54 × 10^− 6^) *DIP2C* (rs61831032, P = 1.06 × 10^− 8^, adjusted *P* = 1.27 × 10^− 6^) were observed by GWEIS for anxiety; *LRRFIP1* (rs62194228, *P* = 6.02 × 10^− 10^; rs3806505, *P* = 7.57 × 10^− 10^; rs55874185, *P* = 7.62 × 10^− 10^) and *ANPEP* (rs41276918, *P* = 1.31 × 10^− 8^) were observed by GWEIS for self-harm, but limited epidemiological and biological evidence illustrated their potential effects. Therefore, further studies are needed to confirm our findings and clarify the potential roles of novel genetic variants in the pathogenesis of mental health.

In contrast with GWAS, GWEIS discovered some novel genes that might influence the risk of anxiety and self-harm. Previous studies focused on the genetic effect on mental disorders, less studies have assessed the interaction role of genes and environment on the mechanism of these complex diseases. Our study demonstrated the interactive association between birth by CS and anxiety, and self-harm. As far as we know, this is the first systemic study exploring the effect of birth by CS as an environmental factor on mental disorders for adults. Our study holds great potential for clarifying the functional relevance of birth by CS with mental disorders and provides novel clues for the pathogenesis of those mental disorders.

However, some limitations of this study should be noted. Like GWAS, some significant SNPs found by GWEIS are located in non-coding regions, which still poses challenges for us to better illustrate our results. Compared with the general population, participants recruited in UK Biobank are healthier and live in the poorer socioeconomic areas [49], called a healthy volunteer bias. Besides, because socioeconomic deprivation is associated with worse mental health [50–52], future studies to explore the effects of socioeconomic deprivation on the mental health of adults that birth by CS are warranted. Other factors such as history of illness, family history of mental illness, exposure to traumatic events, life satisfaction should be considered to eliminate their influence on anxiety and self-harm. Due to the small sample of participants birth by CS in UK biobank which may impact the findings, further researches using larger sample size, different genetic populations are needed to validate the findings of our study. All subjects in this study are of European ancestry. Therefore, it should be careful to apply our study results to other ethnic groups. Furthermore, more experimental studies are needed to validate the novel identified genes to illustrate the biological functions and mechanisms.

## Conclusions

In summary, we observed significant associations between birth by CS and the risk of adult anxiety and self-harm using UK Biobank cohort. GWEIS analysis identified multiple candidate genes which may serve as the underlying genetic mechanisms of the observed association. These findings highlight the importance of policies and strategies to prevent unnecessary CS.

## Electronic supplementary material

Below is the link to the electronic supplementary material.

Supplementary Material 1: Supplementary Table 1. Association between anxiety, self-harm behavior and age and sex, respectively

Supplementary Material 2: Supplementary Figure 1. LocusZoom plot of anxiety associations with birth by caesarean section

Supplementary Material 3: Supplementary Figure 2. LocusZoom plot of self-harm associations with birth by caesarean section

## Data Availability

The datasets used and/or analysed during the current study are available from the corresponding author on reasonable request.

## Abbreviations

CS: Caesarian Section
GWEIS: Genome-wide by Environment Interaction Study
OR: Odds Ratio
YLDs: Years Lived with Disability
DALYs: Disability-adjusted Life-years
WHO: World Health Organization
PHQ-9: Patient Health Questionnaire
GAD-7: General Anxiety Disorder
CI: Confidence Intervals
MAF: Minor Allele Frequencies
ATXN1: Ataxin-1
SCA1: Spinocerebellar Ataxia Type 1
