# Supplementary material for: Association between birth by caesarian section and anxiety, self-harm: a gene-environment interaction study using UK Biobank data
Source: BMC Psychiatry. 2023 Apr 7;23:237. doi: 10.1186/s12888-023-04720-0 (PMC10080817; doi:10.1186/s12888-023-04720-0)
Supplement: Supplementary file 1 — Supplementary Material 1: Supplementary Table 1. Association between anxiety, self-harm behavior and age and sex, respectively [file 12888_2023_4720_MOESM1_ESM.docx]

**Additional file 1: Supplementary Table 1. Association between anxiety, self-harm behavior and age and sex, respectively**

| **Instrument** | **Outcome** | **Beta/ OR (95% CI)** | **SE** | **Statistic** | ***P*** |
| --- | --- | --- | --- | --- | --- |
| Age | Anxiety | -0.020 (-0.022~-0.018) | 0.001 | -16.317 | < 0.0001 |
| Sex |  | 0.545 (0.525-0.567) | 0.019 | -30.923 | < 0.0001 |
| Age | Self-harm | -0.055(-0.057~-0.053) | 0.001 | -45.094 | < 0.0001 |
| Sex |  | 0.638 (0.614-0.663) | 0.019 | -23.120 | < 0.0001 |

Note: OR, odd ratios; CI, confidence interval; SE, standard error. T-test was used to detect age imbalance between case and control group and Chi-square test was used to detect sex imbalance between case and control group.
