# Supplementary material for: Association between birth by caesarian section and anxiety, self-harm: a gene-environment interaction study using UK Biobank data
Source: BMC Psychiatry. 2023 Apr 7;23:237. doi: 10.1186/s12888-023-04720-0 (PMC10080817; doi:10.1186/s12888-023-04720-0)
Supplement: Supplementary file 2 — Supplementary Material 2: Supplementary Figure 1. LocusZoom plot of anxiety associations with birth by caesarean section [file 12888_2023_4720_MOESM2_ESM.pdf]

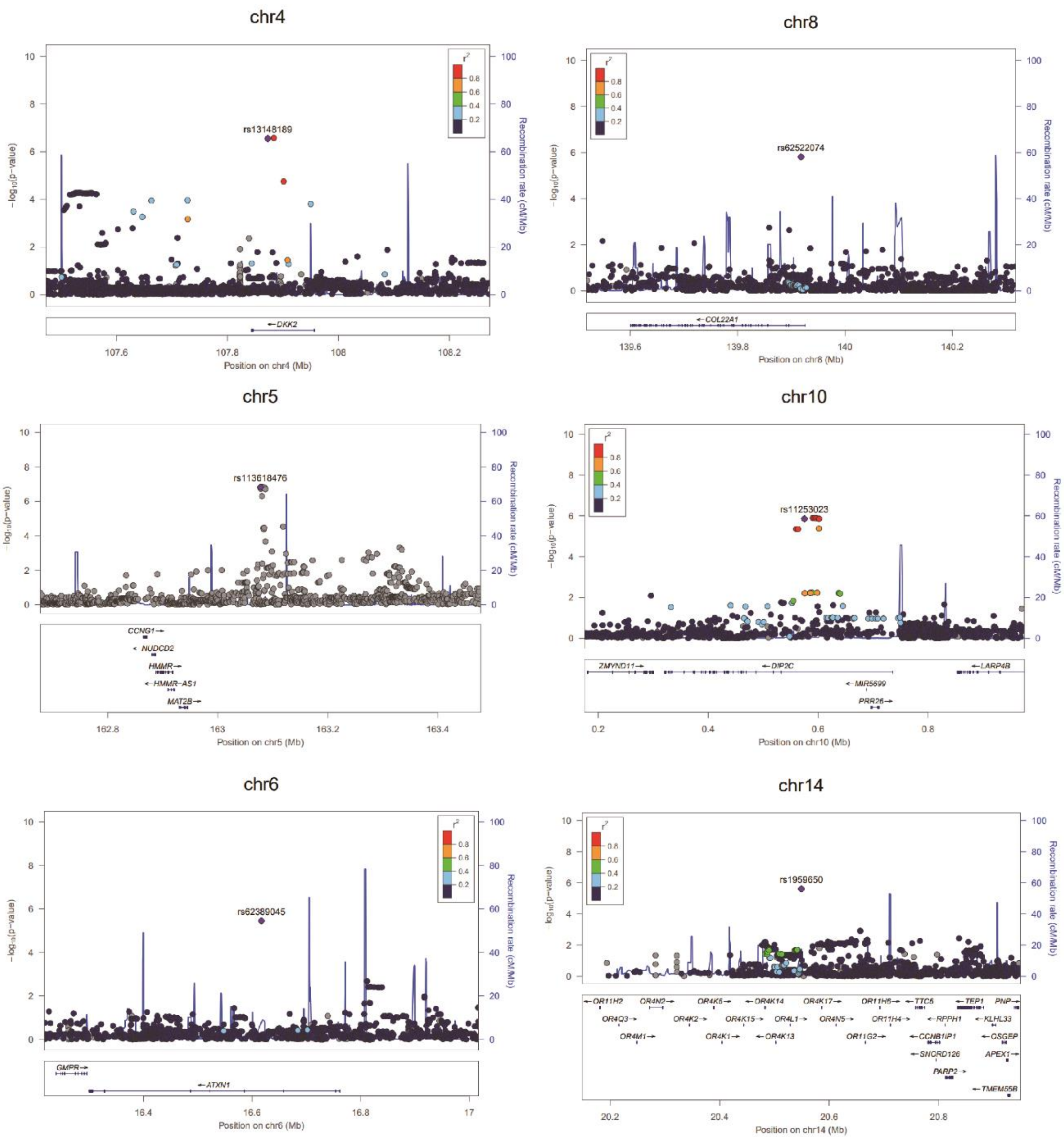

Additional file 2: Supplementary Figure 1. LocusZoom plot of anxiety associations with birth by caesarean section. The plot displays -log10 *P*-value on the y-axis and physical position on the x-axis. The blue line indicates pre-calculated recombination rates (in cM/Mb) at each position. Purple diamond indicates SNP at the locus with the strongest association evidence. Each point represents a SNP. Chr, chromosome.
