# Supplementary figures and images for: Association between birth by caesarian section and anxiety, self-harm: a gene-environment interaction study using UK Biobank data
Source: BMC Psychiatry. 2023 Apr 7;23:237. doi: 10.1186/s12888-023-04720-0 (PMC10080817; doi:10.1186/s12888-023-04720-0)

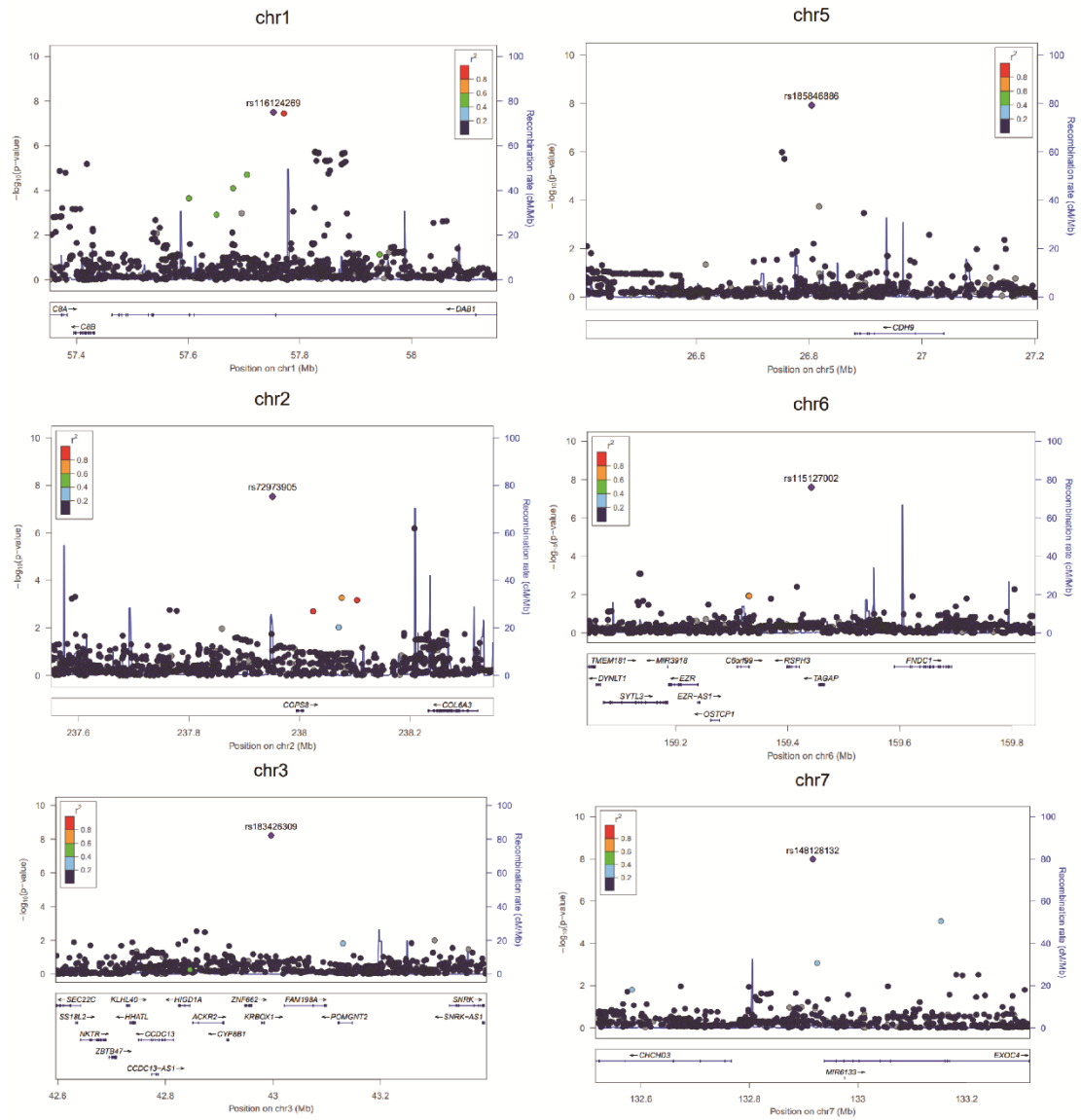

(Continued)

Supplement: Supplementary file 3 — Supplementary Material 3: Supplementary Figure 2. LocusZoom plot of self-harm associations with birth by caesarean section [file 12888_2023_4720_MOESM3_ESM.pdf]
